# Impact of Chest Radiography for Children with Lower Respiratory Tract Infection: A Propensity Score Approach

**DOI:** 10.1371/journal.pone.0096189

**Published:** 2014-05-02

**Authors:** Emmanuelle Ecochard-Dugelay, Muriel Beliah, Caroline Boisson, Francis Perreaux, Jocelyne de Laveaucoupet, Philippe Labrune, Ralph Epaud, Hubert Ducou-Lepointe, Jean Bouyer, Vincent Gajdos

**Affiliations:** 1 Inserm, CESP Centre for research in Epidemiology and Population Health, U1018, Reproduction and Child Development Team, Le Kremlin Bicêtre, France; 2 APHP, Paediatric Department, Hopital Antoine Béclère, Clamart, France; 3 APHP, Radiology Department, Hopital Antoine Béclère, Clamart, France; 4 APHP, Paediatric Department, Centre Hospitalier Intercommunal, Créteil, France; 5 APHP, Paediatric Radiology Department, Hôpital Trousseau, Paris, France; 6 Université Paris Sud 11, Kremlin Bicêtre, France; 7 Université Paris Est, Créteil, Val de Marne, France; 8 Université Paris 6, Paris, France; University ofTennessee Health Science Center, United States of America

## Abstract

**Background:**

Management of acute respiratory tract infection varies substantially despite this being a condition frequently encountered in pediatric emergency departments. Previous studies have suggested that the use of antibiotics was higher when chest radiography was performed. However none of these analyses had considered the inherent indication bias of observational studies.

**Objective:**

The aim of this work was to assess the relationship between performing chest radiography and prescribing antibiotics using a propensity score analysis to address the indication bias due to non-random radiography assignment.

**Methods:**

We conducted a prospective study of 697 children younger than 2 years of age who presented during the winter months of 2006–2007 for suspicion of respiratory tract infection at the Pediatric Emergency Department of an urban general hospital in France (Paris suburb). We first determined the individual propensity score (probability of having a chest radiography according to baseline characteristics). Then we assessed the relation between radiography and antibiotic prescription using two methods: adjustment and matching on the propensity score.

**Results:**

We found that performing a chest radiography lead to more frequent antibiotic prescription that may be expressed as OR = 2.3, CI [1.3–4.1], or as an increased use of antibiotics of 18.6% [0.08–0.29] in the group undergoing chest radiography.

**Conclusion:**

Chest radiography has a significant impact on the management of infants admitted for suspicion of respiratory tract infection in a pediatric emergency department and may lead to unnecessary administration of antibiotics.

## Introduction

Lower respiratory tract infection (LRTI) is one of the most common conditions encountered in pediatric emergency departments (PED) during the winter months, and its management consumes substantial health-care resources.[1] There is substantial diversity in the management of LRTI reflecting the absence of a consensual treatment. Although combinations of clinical features have been proposed to distinguish viral (the most frequent cause) from bacterial diseases, none are sufficiently sensitive to be reliable or widely used.[2]–[4]. Prescription of chest radiography (CR) is common despite recent World Health Organization guidelines advising against its routine use.[5]

Although radiographic findings are often nonspecific, observational studies have reported a higher prevalence of prescription of antibiotics among children in whom CR has been performed.[6], [7] However, the effects of CR may have been overestimated in these reports owing to a likely indication bias, as these children had not been randomly selected for radiography. To avoid such a bias, Rosenbaum and Rubin suggested using the *propensity score* (PS), defined as the probability of receiving a particular treatment or procedure conditional on the observed covariates: two subjects from the two groups compared (CR or no CR) with the same propensity score can be considered as if they had been randomly assigned to the two groups.[8]

The aim of this study was to assess the effects of CR on the likelihood of antibiotic therapy, using PS-based methods to minimize bias due to non-random radiography assignment.

## Patients and Methods

### Study design and clinical data collection

The ethics committee, Comité de Protection des Personnes Ile de France 3, reviewed this project and concluded that this research conformed to generally accepted scientific principles, and to French laws and regulations Informed oral consent was obtained from parents and refusal to participate did not alter the clinical care provided to the patients.

We conducted a prospective study in the PED of an urban general hospital in France (Paris suburb). All infants (less than 2 years of age) who presented for suspicion of LRTI between October 2006 and February 2007 were included. Suspicion of LRTI was defined as one or more of the following clinical findings: hypoxia (<95%), age-related tachypnea, signs of respiratory distress (retractions, nasal flaring, abdominal breathing) or abnormal breath sounds (wheezing, prolonged expiratory phase, or crackles) obtained by auscultation.

Physicians filled in a short questionnaire with a fixed-choice format, collecting information about demographics, case history, physical examination and management. Demographic data included age, gender, history of prematurity, known chronic illness, and previous wheezing episodes. Case history information included duration of symptoms, history of fever, feeding difficulties, and antibiotic use before PED admission. Physicians were also asked to report temperature at first evaluation, respiratory rate, oxygen saturation, retraction signs, auscultation findings, presence of acute otitis media, conjunctivitis or apparent toxicity. They also recorded the following details of the management: CR, antibiotic prescription, and decision about hospitalization. Patients who received antibiotics before admission were excluded from the study because prior antibiotic therapy undoubtedly affects final treatment decision. CR were interpreted by the emergency pediatrician.

### Statistical methods

Descriptive and multivariate data analyses were performed with Stata 11.2 software (Statacorp. 2009. Stata Statistical Software. Release 11. College Station, TX: Statacorp LP).As recommended, the propensity score (PS), i.e. the probability to undergo CR, was calculated with a non-parsimonious multivariate logistic regression model (without an algorithm of selection of variables) with baseline characteristics at admission as independent covariates (14 variables included) and CR as the dependent dichotomized variable. These baseline characteristics were chosen because they were related to the prescription of both CR and antibiotics.[9] Some were dichotomous variables (history of prematurity, infantile asthma, presence of eating disorders, prolonged expiratory phase, crackles, wheezing, conjunctivitis, toxic appearance). Five quantitative variables were transformed into clinically relevant classes (temperature, oxygen saturation, duration of respiratory symptoms, respiratory rate from Liu classification score (*table 1*) and respiratory distress score by Silverman).[10], [11] Three variables were left in continuous form. We studied their relationship with the variable of interest to choose from include linear form (arterial oxygen saturation temperature) or with fractional polynomials (age).[12] The area under the Receiver Operator Characteristic (ROC) curve was calculated to assess model discrimination.[13]

**Table 1:** Age-related respiratory rate, from Liu et al. [10].

	Respiratory rate (/min)		
	1 point	2 points	3 points
Age < 2 months	≤60	61–69	≥70
Age 2 – 12 months	≤50	51–59	≥60
Age 12 – 24 months	≤40	41–44	≥45

To study the association between CR and antibiotic prescription, and controlling for indication bias, we used two methods: adjustment for and matching on PS.[14]

The adjustment method consisted of including PS as a continuous adjustment variable in a logistic model with antibiotic prescription as the dependent variable and CR as an independent variable. To ensure that confounders are taken into account as best as possible, we also adjusted on a subset of nine covariates linked to antibiotic prescription, leading to a *double-robust* model as described by D’Agostino and al.[15] This method provided an odds-ratio (OR) for CR with 95% confidence interval (CI). Variables related to antibiotics prescription were selected by a stepwise procedure: fever, blood oxygen saturation, duration of respiratory symptoms, age, prematurity, prolonged expiratory phase, presence of crackles, conjunctivitis, toxic appearance.

The matching method consisted of matching two subjects on PS (one from each group, CR or no CR) so as to obtain pairs with the same set of baseline covariates.[16] We used a nearest-neighbor matching method that matched pairs of subjects with the nearest PS value. This method provided the difference between percentages of the two groups for which antibiotics were prescribed (with the 95% CI), after taking into account indication bias.

Missing data (less than 5% per variable) were imputed using a method based on multiple chained equations.[17]

## Results

### Study population

Demographic and clinical characteristics of the sample (*n* = 697) are shown in *table 2*. The mean age was 7.7 months (+/−5.8). Prematurity and a history of infant asthma were reported in 14% f and 21% of patients, respectively. Moderate respiratory distress was observed in 56% of the infants (respiratory distress score of 1), and 14% presented with more severe distress (score ≥2). In half of the patients, the duration of respiratory symptoms was short (less than 3 days) and 57% had a normal age-related respiratory rate. Most children (87.4%) had no hypoxia (oxygen saturation ≥95%). The mean observed temperature was 37.9°C (+/− 1). At triage, 58%, 9% and 48% of subjects presented wheezing, crackles and prolonged expiratory phase, respectively. Fifty-one percent of patients underwent CR. Thirty-one CR were considered to be abnormal (8.7%): 29 with alveolar condensation, one with lobar atelectasia and one with cardiomegaly. Antibiotics were prescribed in 31% of patients in the group with CR, and 8% of the group without CR.

**Table 2:** Demographics of the study population (*n* = 697).

Characteristics	All	CR group	no CR group
		n = 355	n = 342
Age, *N* (%), months			
≤ 3 m	160 (23.0)	99 (27.9)	61 (17,8)
> 3 m	537 (77.0)	256 (71.1)	281 (82.2)
Age, mean (se), m	7.7 (5.8)	6.4 (6.1)	6.9 (5.3)
Infant asthma (≥3 bronchiolitis), *N* (%)	143 (21.1)	75 (21.1)	68 (19.9)
Prematurity <37 weeks of gestation, *N* (%)	92 (14.0)	59 (16.6)	33 (9.6)
Duration of respiratory symptoms, mean (se), days	4 (5.4)	4.0 (5.6)	3.9 (5.2)
Duration of respiratory symptoms, *N* (%), days			
[0–2] jours	342 (49.9)	171 (48.9)	171 (51.0)
] 2–4] jours	182 (26.6)	103 (29.4)	79 (23.6)
] 4–30] jours	161 (23.5)	76 (21.7)	85 (25.4)
Feeding difficulties, *N* (%)	249 (36.6)	171 (49.3)	78 (23.4)
Maximum of temperature, mean (se), °C	37.9 (1.0)	38.1 (1.0)	37.6 (0.8)
Age-related respiratory rate (Liu (9)), *N* (%)			
1 point	366 (57.4)	157 (48.5)	209 (66.8)
2 points	89 (14.0)	48 (14.8)	41 (13.1)
3 points	182 (28.6)	119 (36.7)	63 (20.1)
Oxygen saturation, *N* (%)			
≥95%	587 (87.4)	270 (78.2)	317 (96.4)
[92–94]	52 (7.7)	43 (12.5)	9 (2.7)
<92	33 (4.9)	30 (8.8)	3 (0.9)
Respiratory distress score (Silverman), *N* (%)			
0	204 (30.0)	65 (18.9)	139 (41.5)
1	381 (56.0)	207 (60.0)	174 (51.9)
2	71 (10.4)	54 (15.6)	17 (5.1)
3	24 (3.5)	19 (5.5)	5 (1.5)
Wheezing, *N* (%)	401 (58.1)	202 (57.5)	199 (58.7)
Crackles, *N* (%)	63 (9.1)	59 (16.8)	4 (1.2)
Prolonged expiratory phase, *N* (%)	330 (47.8)	174 (49.6)	156 (46.0)
Otitis	62 (8.9)	34 (9.6)	28 (8.2)
Conjunctivitis, *N* (%)	25 (3.7)	13 (3.7)	12 (3.6)
Apparent toxicity, *N* (%)	27 (3.9)	6 (1.7)	21 (6.0)
Abnormal chest X-ray	31 (8.7)	31 (8.7)	-
alveolar condensation	29 (8.4)	29 (8.4)	-
lobar or segmental atelectasia	1(0.3)	1 (0.3)	-
Cardiomegaly	1(0.3)	1 (0.3)	-
Antibiotics, *N* (%)	136 (19.5)	108 (31.0)	28 (8.4)

### Propensity score

**Figure 1:**
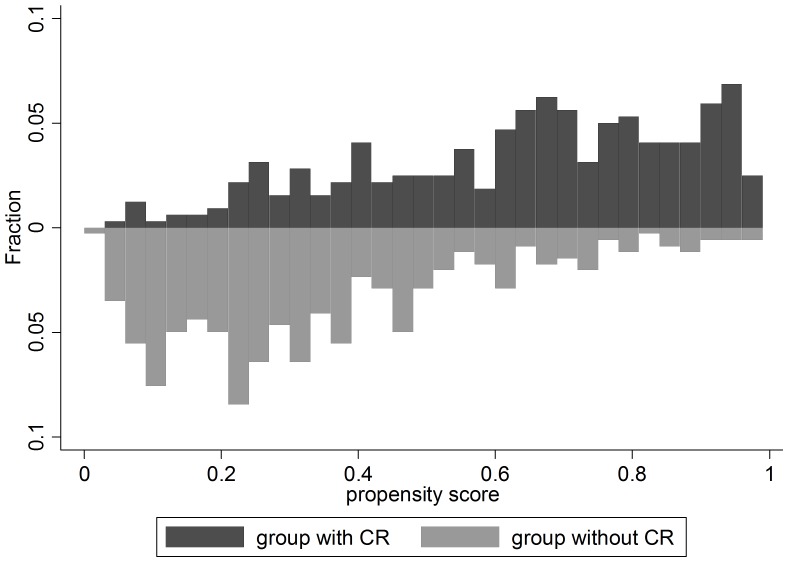
Histograms of propensity score values for the two groups (CR or no CR). The mean propensity score was lower in the group that did not have chest radiography, although there was a wide overlap between both groups.

Distributions of PS are displayed by group (CR or no CR) in *figure 1*. The mean PS was lower in the group without CR than in the group with CR. However, there was a wide overlapping area between distributions of both groups, thus allowing a high-quality matching. The area under ROC curve for propensity score was 0.84 [0.83–0.85], indicating a good discrimination capacity.

### Effect of undergoing chest radiography on antibiotic use *(* *table 3* *)*

**Table 3:** Effect of chest radiography on antibiotic use.

**Crude odds-ratio** (OR, 95%CI, p-value)	OR = 4.9; [3.1–7.8]; p<0.001
**Adjustment method** (OR, 95%CI, p-value)	OR = 2.3; [1.3–4.1]; p = 0.004
**Matching method** (percentage, 95%CI)	0.186; [0.08–0.29]

The crude OR between CR and antibiotic prescription was 4.9 (95CI [3.1–7.8], p<0.001).

After adjustment for PS and for the subset of nine covariates linked to antibiotic therapy, the association between CR and antibiotics prescription remained significant: OR 2.3 (95%CI [1.3–4.1], p = 0.004).

With the matching method, we found a significantly higher rate (18.6% higher) of antibiotic prescription in the group of patients with than in the group without CR (mean effect = 0.186, 95CI [0.08–0.29]).

## Discussion

Our study showed that children presenting with LRTI to an emergency department were more likely to receive antibiotics if they underwent CR as part if their initial evaluation. Using PS, this association remained statistically significant after taking into account a possible indication bias. Indeed, antibiotics were prescribed in 18.6% more o infants with CR than in those without CR. This finding suggested that the management of two infants with identical LRTI prognostic baseline characteristics differed, depending on whether or not they had CR: therefore CR had a significant effect on care and might have lead to unnecessary antibiotic therapy. For the purpose of this study, CR images were independently reviewed by two radiologists who were blinded for clinical history. They were simply aware that the imaging was obtained as part of LRTI evaluation but were blinded to other clinical history and outcomes. The Emergency Department practitioners made clinical decisions based on their own interpretation of the CR. This was very important because one of the aim of our study was to determine if the prescription of CR influenced antibiotic prescription, especially without interpretation by a pediatric radiologist.

Our findings are consistent with previous studies. A randomized control trial published in 1998 reported that performing CR had consequences for both management and clinical outcome in 522 children who met the WHO case definition of pneumonia.[18] CR was randomly allocated. Although it was not the primary outcome measure in the study, antibiotic use was higher in the CR group (60.8% vs. 52.2%, p = 0.05). However, children with clinical signs of severity were excluded from this previous study, limiting the extrapolation of the results to LRTI of other causes. Other studies used observational data and did not consider indication bias. Shuh et al. studied 265 infants with typical bronchiolitis who underwent CR.[7] They reported more frequent prescription of antibiotics after CR (post-radiography scenario), relative to the PED physician's intention to treat before CR (pre-radiography scenario), with a percentage difference of 12 (95%CI [0.08–0.16]). However, asking the question may itself have affected physicians' responses before and after CR. Another study [6] showed that performing CR was associated with an increased likelihood of antibiotic use among children over 3 months old (OR 1.22, 95%CI [1.10–1.36], p<0.001); for children less than 3-months-old, there was a similar but not significant relationship (OR 1.11, 95%CI [0.96–1.28]).

Our study had two main strengths: the choice of the study population and the control of indication bias using PS. We analyzed a large sample of infants under two years with various clinical forms and severity scores of respiratory distress. Our study population was not selected, so it was likely to represent the population encountered in clinical practice. We focused on infants because clinical forms and causes of respiratory tract infections in children under 2 years of age are generally homogeneous and comparable. We had a CR rate of 51% that was consistent with rates reported in previous observational studies [6], [19]–[21].

Using PS (i.e. the probability that a patient would have a CR) to adjust the association between CR and antibiotic use, we mimicked a “quasi-randomized” experiment in which two subjects with the same baseline characteristics are randomly assigned to two groups (CR or no CR). It can be shown that PS provides an unbiased estimate of the association between CR and antibiotic use, if all confounders are considered.[22] Therefore, we collected data for numerous baseline covariates and built PS according to current guidelines recommending the creation of a non parsimonious model to use all available data.[23], [24] The continuous variables were modeled according to their linear gradient, or as categorical. All these considerations lead to a robust method for estimating the independent association between CR and antibiotic prescription from observational data.

There were several potential limitations to our study. Some relevant factors may have been unobserved or unmeasured. Consequently, our results were not as reliable as those of a randomized trial. Data were collected in only one PED such that extrapolation of the results to other centers or to infants consulting in private practice may be questionable. We also observed a higher rate of antibiotic use in the group that underwent CR (30.9%) than in another previous study reporting antibiotic therapy in 15% of the CR group.[7] However, the total rate of antibiotic therapy was low (20%), compared to values reported in the literature (between 30 and 60%).[6]

Finally, we stressed that CR prescriptions must be limited for several reasons, other than reducing the rate of unnecessary antibiotic therapy. Although the radiation dose associated with CR is small (0.02 mSv, to be compared to natural exposure estimated to be about 0.05 mSv per week), a recent report on exposure of the French population to ionizing radiation related to acts of medical diagnosis reported a mean of 0.2 CR per year per child under one year old (approximately 160,000 procedures per year for a country with 800,000 births annually).[25] Furthermore, in a cost-effectiveness analysis, Yong et al. concluded that omission of routine CR in infants with bronchiolitis saved $59 per patient (due to savings in radiography and hospitalization costs) without compromising diagnostic accuracy of alternate diagnosis and associated pneumonia.[26] However, in some particular situations (ie when clinical signs are difficult to be interpreted), CR may be essential in order to eliminate bacterial infection requiring antibiotics.

## Conclusion

Our study confirmed the previously observed increase of antibiotic use among children undergoing CR, even when indication bias was taken into account. We can assume that part of this antibiotherapy was unnecessary, in the context of an emergency room. Guidelines should be updated to reduce CR prescriptions in PED for children with suspected LRTI.
